# A universal self-charging system driven by random biomechanical energy for sustainable operation of mobile electronics

**DOI:** 10.1038/ncomms9975

**Published:** 2015-12-11

**Authors:** Simiao Niu, Xiaofeng Wang, Fang Yi, Yu Sheng Zhou, Zhong Lin Wang

**Affiliations:** 1 School of Materials Science and Engineering, Georgia Institute of Technology, Atlanta, Georgia 30332, USA; 2 Department of Precision Instrument, Tsinghua University, Beijing 100084, China; 3 Beijing Institute of Nanoenergy and Nanosystems, Chinese Academy of Sciences, Beijing 100083, China

## Abstract

Human biomechanical energy is characterized by fluctuating amplitudes and variable low frequency, and an effective utilization of such energy cannot be achieved by classical energy-harvesting technologies. Here we report a high-efficient self-charging power system for sustainable operation of mobile electronics exploiting exclusively human biomechanical energy, which consists of a high-output triboelectric nanogenerator, a power management circuit to convert the random a.c. energy to d.c. electricity at 60% efficiency, and an energy storage device. With palm tapping as the only energy source, this power unit provides a continuous d.c. electricity of 1.044 mW (7.34 W m^−3^) in a regulated and managed manner. This self-charging unit can be universally applied as a standard ‘infinite-lifetime' power source for continuously driving numerous conventional electronics, such as thermometers, electrocardiograph system, pedometers, wearable watches, scientific calculators and wireless radio-frequency communication system, which indicates the immediate and broad applications in personal sensor systems and internet of things.

## Abstract (web summary)

Triboelectric nanogenerators utilize triboelectrification for harvesting energy from ambient mechanical motions. Here, the authors report an integrated triboelectric nanogenerator system using only human biomechanical energy to generate mW-level DC electricity that is enough to continuously drive various commercial mobile electronics.

## Graphical abstract

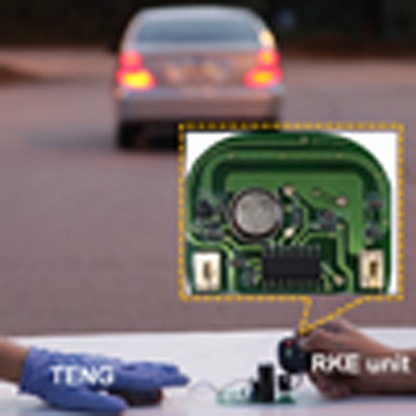

A rapid development in personal electronics and sensor networks^1^,^2^ has raised urgent and challenging requirements for their portable and sustainable power sources, which are now mostly using batteries. In many modern mobile electronics and internet of things, batteries have become the largest and heaviest component of the entire unit, and they are required to be charged periodically due to limited lifetime, leading to an inevitable design trade-off dilemma between mobility (size and weight) and sustainability (lifetime). Although this dilemma can be eased to some extent with the development of high-energy-density materials^3^,^4^,^5^, it is not solved from the root. A fundamental solution to this challenge is to develop technologies that can constantly convert ambient energy into electricity and get the battery continuously charged to ensure its sustainable and maintenance-free operation^6^,^7^,^8^. Ambient energy sources exist in various forms, such as mechanical, thermal^9^,^10^, chemical^11^ and solar^12^,^13^. Distinguished from others, mechanical energy is the one that is available almost everywhere and at any time, to name a few, gentle airflow, ambient sound, vibration, human body motion, ocean waves and so on. Although this type of energy could be converted by using traditional generators based on electromagnetic induction, they are usually heavy in weight and large in size. More importantly, their designs are rigid and cannot be easily adapted for harvesting body motion energy and vibration energy due to their intrinsic structure complexity^14^,^15^,^16^,^17^. Finally, as limited by the working mechanism, an electromagnetic generator usually produces a small output voltage (<1 V) when its size is small, so that the output power cannot be effectively utilized because of the threshold voltage required to overcome a diode to function in rectification. Alternatively, micro-electromechanical-based energy harvesting using electrostatic or piezoelectric effect commonly provides a power level of ∼μW (refs 18, 19, 20, 21, 22, 23), so it can hardly meet the needs for wearable and portable electronics that require mW–W level of power.

Contact electrification, known for thousands of years, is about charge transfer between two materials when they contact with each other, but this effect is rarely utilized for energy harvesting until recently^24^,^25^. Triboelectric nanogenerators (TENGs) were invented by incorporating contact electrification and electrostatic induction, and it has been demonstrated as a promising technology with numerous advantages, including large power density, high efficiency, diverse choice of materials for fabrication, low cost and lightweight^26^,^27^,^28^,^29^. TENG has various working modes that cover almost all of the mechanical motions in our daily life. Besides, it can be made into thin-film structure with high compactness and three-dimensional integration scalability that can potentially lead to mW–W output power. However, to embed TENGs into electronic system for practical applications, there are major challenges to overcome. First, due to the randomness of mechanical energy source and the intrinsic capacitive behaviour of TENGs, the output of a TENG normally is in the form of pulsed a.c. signal with variable frequency^30^. Second, TENG usually has a high output voltage in the order of hundreds of volts but low current in the order of microamps, which has a huge mismatch with the requirement of mobile electronics. Besides, the high inherent impedance of TENGs results in ultralow energy conversion efficiency using conventional transformer for power management. Third, owing to the a.c. output, TENGs cannot be taken as steady power sources for directly driving electronics. Therefore, an energy storage unit is necessary to serve as a ‘reservoir' for collecting the generated charges and outputting them in a managed and regulated manner. However, a direct integration of a TENG with an energy storage device has shown extremely low charging efficiency and large power loss due to the huge impedance mismatch between TENGs and energy storage units, where TENGs have inherently high impedance while energy storage devices such as batteries and capacitors usually have low impedance^31^,^32^. Therefore, although there are several TENG-based ‘self-powered' systems being reported, all of these systems either cannot work continuously due to the low level of generated d.c. power or the entire system cannot be exclusively driven by the harvested energy if the data measurement and processing systems as well as data transmission components are included.

Here we present a genuine self-powered system to meet mW requirement of personal electronics by solving the challenges presented above. The system includes a TENG, a power management circuit and a low-leakage energy storage device. We perform system-level optimization to ensure the collaborative work of all the system components. Especially, a power management circuit is designed to solve the impedance mismatch problem, which can achieve 90% board efficiency and 60% total efficiency, about two orders of magnitude improvement compared with direct charging. Driven by palm tapping, this power unit can provide a continuous d.c. electricity of 1.044 mW on average power in a regulated and managed manner that can be universally applied as a standard power source for continuously driving numerous conventional electronics, such as a thermometer, a heart rate monitor (electrocardiograph (ECG) system), a pedometer, a wearable electronic watch, a scientific calculator and a wireless radio-frequency communication system. Our study demonstrates the first power unit that utilizes widely accessible biomechanical energy source to sustainably drive a broad range of commercial mobile and wearable electronic devices.

## Results

### Design of the system framework

**Figure 1:**
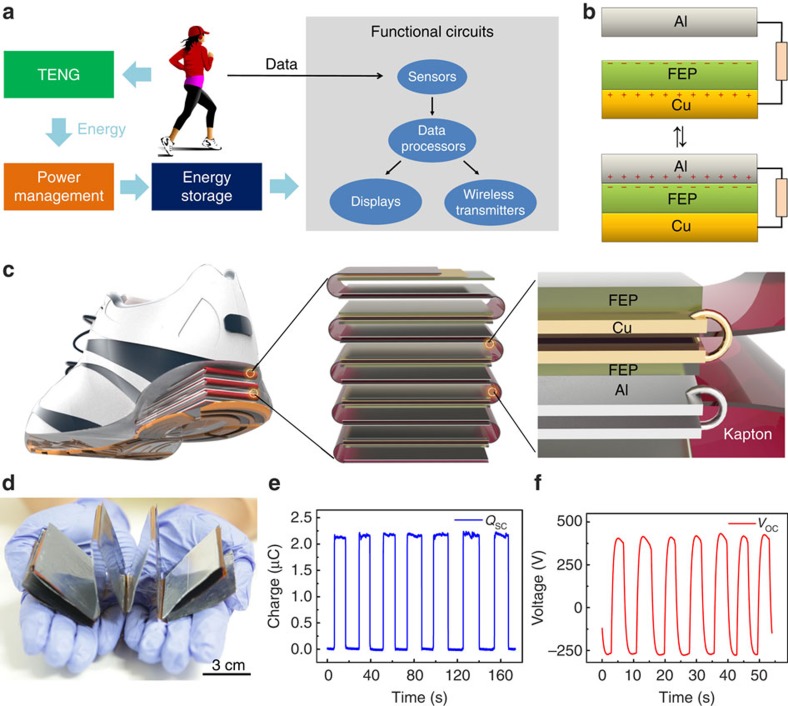
Structure design of the self-powered system. (**a**) System diagram of a TENG-based self-powered system. (**b**) Working mechanism of an attached-electrode contact-mode TENG. (**c**) Structure of the designed multilayer TENG. (**d**) Photo of an as-fabricated TENG. (**e**) Short-circuit current output and (**f**) open-circuit voltage output of the as-fabricated TENG.

This integrated human-motion-driven self-powered system is shown in Fig. 1, including a human-motion-powered self-charging power unit and functional circuits for different applications. The TENG scavenges the human biomechanical energy and converts it to a.c. electricity. Then this a.c. electricity is converted to a d.c. electric output that matches to the input of the energy storage unit by the power management circuit. Finally, the energy storage unit provides d.c. electricity to the whole functional circuits, including sensors, data processors, displays and wireless transmitters. The signals from sensors and data processors are either visualized on a LCD displayer or sent out remotely through a wireless transmitter.

### Design of the multilayered TENG

The TENG is first designed and optimized to efficiently harvest human biomechanical energy. We choose a multilayered attached-electrode contact-mode TENG to effectively collect the energy from human walking and running. The basic working principle of attached-electrode contact-mode TENGs is shown in Fig. 1b, which utilizes the conjugation between contact electrification and electrostatic induction^33^. As shown in Fig. 1c, a Kapton film is shaped into a zigzag structure with 10–15 layers^34^. For each layer, a thin aluminium foil and fluorinated ethylene propylene (FEP) layer are utilized as the triboelectric materials. The Al foil also serves as one electrode. Copper is evaporated at the backside of the FEP layer as the other electrode. As shown in Fig. 1d, the as-fabricated TENG has small volume and lightweight (5.7 × 5.2 × 1.6 cm/29.9 g for a 10-layer TENG and 5.7 × 5.2 × 2.4 cm/43.6 g for a 15-layer TENG). The optimum thickness of FEP for this design is 125 μm according to theoretical calculation and structural design considerations (Supplementary Fig. 1). Besides, nanostructures are created on the surface of FEP and Al foil to enhance the device performance (Supplementary Fig. 2)^35^. Embedded in the shoe insoles, a human walking can drive this TENG to generate about 2.2 μC short-circuit transferred charge and about 700 V voltage output, as shown in Fig. 1e,f. This multilayered TENG is able to work reliably in various kinds of environment. Distinguished from the sliding TENGs, this contact–separation working mechanism ensures little friction and abrasion of the two surfaces during the contact and separation processes, which hugely enhance the durability of this device. As shown in the durability test (Supplementary Fig. 20), the normalized transferred charge does not show obvious degradation even after about 180,000 cycles. Besides, humidity and moisture has also been proven to have little effect to the TENG's performance in a previous study^36^.

### Design of the power management circuit

The key technical challenge is to efficiently store the TENG a.c. electric energy into an energy storage unit. Traditionally, a bridge rectifier is used to convert the a.c. to d.c. and then the energy is directly stored into a large capacitor or a battery (called direct charging)^32^. However, since TENGs have inherently high impedance, such design always faces a huge impedance mismatch especially when the energy source is low-frequency human biomechanical energy, resulting in ultralow energy storage efficiency^31^. For example, if a TENG is used to directly charge an ideal 1-V battery, the theoretical total efficiency *η*_total_ is only about 1% even when the battery's current leakage and internal resistance are neglected (Supplementary Discussion, Section 3.3). Another possible method is to utilize a transformer to match the impedance between the TENG and the energy storage device. However, since TENGs have high internal impedance and pulsed signal output, all practical transformers suffer a huge loss in power conversion, as dictated by their limited quality factor, insufficient primary inductance, poor low-frequency response and finite narrow bandwidth.

Therefore, a new design is required to solve this challenge. From our previous work, when a TENG is utilized to charge a capacitor *C*_temp_ through a bridge rectifier under periodic mechanical motion (initial voltage of *C*_temp_ is 0 V), the voltage of *C*_temp_ (*V*_temp_) has the following relationship with the charging time *t* (ref. 31):


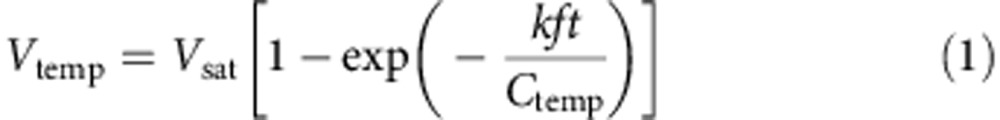


where *V*_sat_ and *k* are parameters determined by the TENG's design, and *f* is the external mechanical motion frequency. Accordingly, the average power (*P*_avg_) stored in *C*_temp_ from time 0 to *t* can be given by:


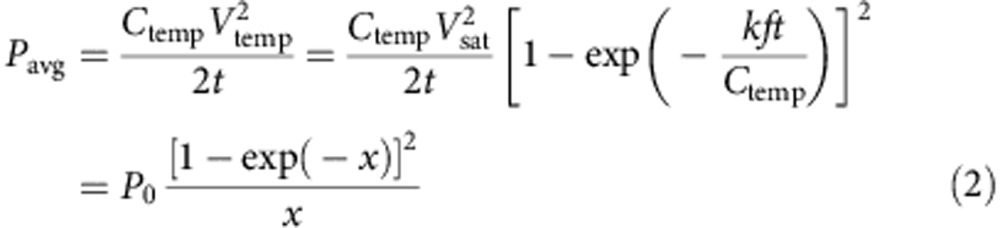


where a dimensionless parameter *x* is defined as *kft*/*C*_temp_ and *P*_0_ is a power constant defined as 
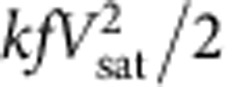
.

**Figure 2:**
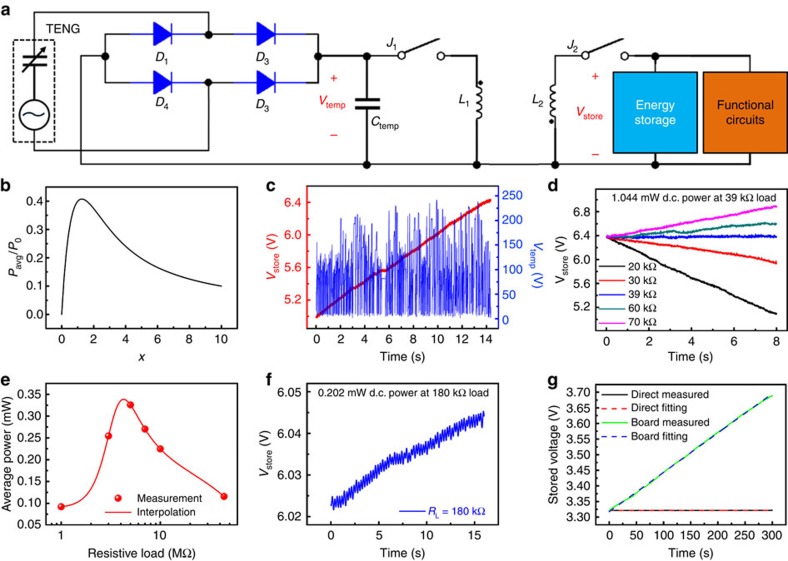
Design of the power management part for regulated TENG outputs. (**a**) Circuit diagram of the power management circuit. (**b**) Theoretical calculation of the optimized charging time to demonstrate the design of the power management circuit. (**c**) Board efficiency measurement results. (**d**) Measurement of the maximum d.c. power of this system driven by human biomechanical energy. (**e**,**f**) Total efficiency measurement results. (**e**) Measurement of the a.c.-harvested power from a resistor. (**f**) Measurement of the d.c.-harvested power from the power management circuit. (**g**) Comparison of the charging current between direct charging and board charging.

As shown in Fig. 2b, there is an optimized *x*_opt_ equal to 1.25643 when the *P*_avg_ reaches its maximum value of 0.4073*P*_0_. At this optimized *x*_opt_ (*t*_opt_), *V*_temp_ reaches 0.7153*V*_sat_, which is the optimized charging voltage *V*_opt_. Note that *V*_opt_ only depends on TENG parameters (independent of *C*_temp_). At this point, *η*_total_ reaches 75.0%, which is the highest charging efficiency for a capacitor charged from 0 V by TENG (Supplementary Discussion, Section 3.4).

With the above theoretical understanding of TENG charging characteristics, we can design the following charging strategy for maximized energy storage efficiency. First, a small temporary capacitor (*C*_temp_) is charged by the TENG from 0 V. Once its voltage reaches *V*_opt_ (impedance match condition is reached), the energy stored in *C*_temp_ begins to be transferred to the final energy storage unit (a large capacitor or a battery) to maximize *η*_total_. When the energy transfer finishes, the voltage of *C*_temp_ drops back to close to 0, and then *C*_temp_ is recharged by the TENG to reach *V*_opt_ again. With this optimized charging cycle, *η*_total_ can theoretically reach 75% (Supplementary Discussion, Section 3.4).

This theoretical charging cycle can be realized by a two-stage power management circuit, as shown in Fig. 2a. At the first stage, a temporary capacitor *C*_temp_ is charged by a TENG through a bridge rectifier. The second stage is for energy transfer from *C*_temp_ to the final energy storage unit. Since transferring electrostatic energy directly from a small capacitor to a large capacitor (or a battery) results in huge energy loss (Supplementary Discussion, Section 4), two automatic electronic switches (controlled by a logic control unit, the power of both switches and their logic control unit is supplied from the final energy storage unit) and a coupled inductor are utilized in the second stage^37^,^38^. The detailed operation process is shown below to achieve efficient energy transfer in this stage. First, both the switches *J*_1_ and *J*_2_ are open to avoid interference of the charging process of *C*_temp_. When *V*_temp_ reaches *V*_opt_, electronic switch *J*_1_ closes. As a result, the energy starts to transfer from *C*_temp_ to inductor *L*_1_ and *V*_temp_ starts to drop. When the energy is thoroughly transferred to *L*_1_, the switch *J*_1_ opens and *J*_2_ closes. As a consequence, the current of *L*_1_ falls to 0 instantaneously. Besides, since the total magnetic flux linkage in the coupled inductance cannot change abruptly, the current of *L*_2_ will suddenly rise up, corresponding to the energy transfer from *L*_1_ to *L*_2_. Finally, the energy stored in *L*_2_ will automatically transfer to the final energy storage unit because of the closure of *J*_2_. When the energy stored in *L*_2_ is thoroughly sent out, *J*_2_ is open again and another charging cycle begins.

### Performance of the power management circuit

The operation of such a power management system is shown in Fig. 2c–g. Figure 2c shows the voltage profiles of both temporary capacitor (*V*_temp_) and final storage capacitor (*V*_store_) when one 15-layer TENG and a 1-mF Al-electrolyte capacitor were utilized as the energy harvester and the energy storage unit (*C*_store_), respectively (Supplementary Fig. 9). While the TENG is driven by palm tapping, *C*_temp_ is charged by the TENG and discharged through the energy transfer network, resulting in an oscillation of *V*_temp_ between 230 and 0 V. During the drop phase of *V*_temp_, *V*_store_ is raised up by the transferred energy from *C*_temp_. Note that if there was no mechanical energy input (from 5.4 to 5.7 s in Fig. 2c), both *V*_temp_ and *V*_store_ will decrease slowly because of the system leakage current and the power consumption of the electronic switches. The performance of the energy transfer network can be evaluated by the board efficiency (*η*_board_), which is defined as the ratio of the total energy stored in *C*_store_ to the total energy transferred out from *C*_temp_. From the data shown in Fig. 2c, the total energy sent out from *C*_temp_ is calculated as 9.160 mJ, while the total energy stored in *C*_store_ is calculated as 8.243 mJ (Supplementary Discussion, Section 5). So this energy transfer network has *η*_board_=90.0%, which is hugely improved in comparison with that of one-step direct energy transfer process (Supplementary Discussion, Section 4).

To measure the d.c. power delivered to the load by the system as driven by palm tapping, a load resistor *R*_L_ is connected in parallel with the storage capacitor (see Supplementary Discussion Section 3.2 for detailed experimental configuration). The storage capacitor *C*_store_ is charged by two 15-layer as-fabricated TENGs and then provides d.c. power for *R*_L_. As shown in Fig. 2d, when *R*_L_ is high, the power consumption of *R*_L_ is lower than the power provided by TENG, so *V*_store_ has a positive slope with time. As the load resistance *R*_L_ decreases, the load power consumption increases and the charging slope of *V*_store_ decreases. When the charging slope reduces to 0 (39 kΩ, Supplementary Fig. 5), the power delivered from palm tapping is the same as the power consumed on the load, which is calculated as 1.044 mW (7.34 W m^−3^). When *R*_L_ continues to decrease (20 kΩ), the power delivered from palm tapping is not enough to compensate the energy consumption on the load, and the charging slope becomes negative.

The most important parameter of the power management circuit is the total efficiency *η*_total_, which is defined as the ratio of the maximum d.c. power stored into the storage unit to the maximum a.c. power delivered to a resistive load (Supplementary Discussion, Section 3). To measure *η*_total_, first the maximum a.c. power delivered to a resistive load can be extracted by the TENG resistance-matching measurement (Supplementary Fig. 8). As shown in Fig. 2e, the maximum a.c. energy generated by TENG is 0.3384, mW at an optimum load resistance of 4.26 MΩ. Second, the maximum d.c. power delivered through the power management board can be measured using the method shown above, which is 0.2023, mW (Fig. 2f) under the same mechanical triggering (from an electric motor). Therefore, *η*_total_ is calculated as 59.8%.

Compared with direct charging, the power management board shows a huge enhancement of the charging efficiency. In another supercapacitor charging experiment (see Supplementary Discussion Section 6 for detailed experimental configuration), the net supercapacitor charging current is 13.82 nA through direct charging and 15.14 μA through the designed power management board, which is improved by as high as 1,096 times! Note that a transformer shows even worse performance than direct charging. As shown in Supplementary Fig. 11, the charging current of a supercapacitor cannot even compensate the leakage current when a 10:1 transformer is utilized as the impedance match network.

### Broad applications as a sustainable d.c. power source

**Figure 3:**
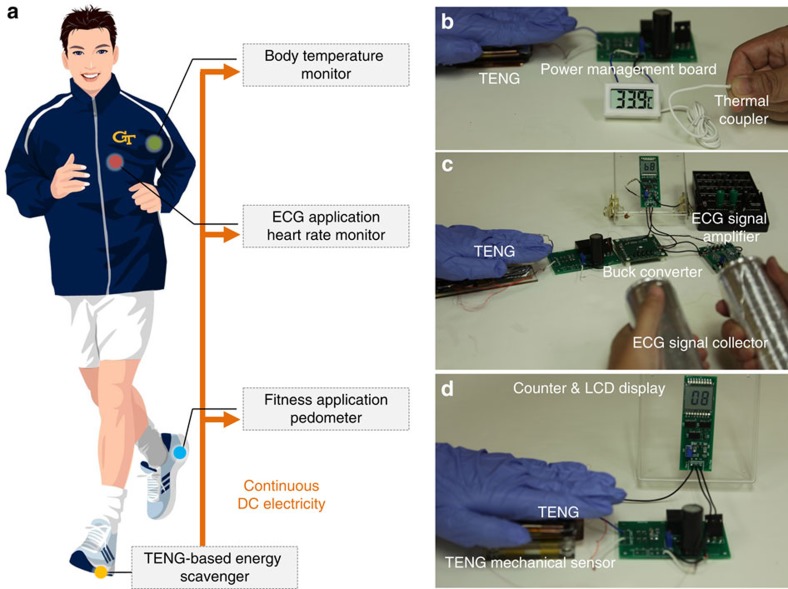
Application in self-powered human activity sensors. (**a**) System configuration of self-powered human activity sensors. (**b**) Demonstration of a self-powered temperature sensor. (**c**) Demonstration of a self-powered heart rate monitor (ECG) system. (**d**) Demonstration of a self-powered pedometer.

This human-motion-charged power unit have wide applications in realizing self-powered human-activity sensors, which makes them self-sufficient without any external power sources. Several applications have been demonstrated by utilizing this self-charged unit to sustainably drive various commercial electronic systems (Fig. 3a). In the first demo, this human-motion-charged power unit was connected to a commercial temperature sensor (Fig. 3b, detailed system diagram is shown in Supplementary Fig. 12). This temperature sensor utilizes a thermal couple to sense the external environment temperature. Then the sensing analogue signal is digitalized through an analogue–digital converter (ADC). Finally, the digital signal is shown on a LCD display to fulfil the visualization of the output. As shown in Supplementary Movie 1, even under very gentle 1.6 Hz palm tapping (palm tapping is utilized mainly for easier filming purpose, other kinds of human motion such as foot tapping is also suitable for the application), this human-motion-charged power unit could supply enough power to maintain continuous operation of the temperature sensor. There were not any external power sources/batteries inside to power any part of the whole functionalized system.

This kind of power unit can also serve as power source for human health monitoring. As a typical example, we realized a self-powered ECG system (Fig. 3c, detailed system diagram shown in Supplementary Fig. 13). The bioelectricity signal across human left hand and right hand was first collected through the ECG signal collector (two conducting Al rods), and then amplified and filtered through an ECG signal amplifier (AD8232, Analog Devices). Such amplified heart rate signal was shown in Supplementary Fig. 14. After that, an ADC was utilized to convert the analogue signal to digital signal, which was then captured by a digital counter. Finally the output of the digital counter was shown on the LCD display to visualize the signal. Besides, a buck converter was utilized to solve the supply voltage mismatch between the ECG signal amplifier (2–3.3 V) and the LCD display (>4.5 V)^37^. As shown in Supplementary Movie 2, the number on the LCD display increased by 1 every time the heart beats. At the same time, the storage capacitor voltage maintained a positive slope, which showed that the power provided from palm tapping was high enough to power the whole ECG system.

This kind of mechanical power source is also capable of being utilized in various fitness applications. For example, a self-powered pedometer system was realized (Fig. 3d, detailed system diagram shown in Supplementary Fig. 15). In this system, the mechanical sensing part was realized by another attached-electrode TENG. When pressure was applied, this attached-electrode TENG could generate a voltage peak, which made this mechanical sensor consume zero electric power. Similar to the above heart rate signal, this voltage peak signal can be captured by an ADC and a digital counter and then visualized on the LCD display. As shown in Supplementary Movie 3 (Supplementary Movie 7 driven by foot tapping), the number on the LCD display increased by 1 every time pressure was applied on the mechanical sensor. Besides, while the pedometer maintained its normal function, *V*_store_ increased quickly from the initial value of 5.02 to 5.67 V. This fast increase is from the ultralow system power consumption due to the utilization of the self-powered TENG mechanical sensor.

**Figure 4:**
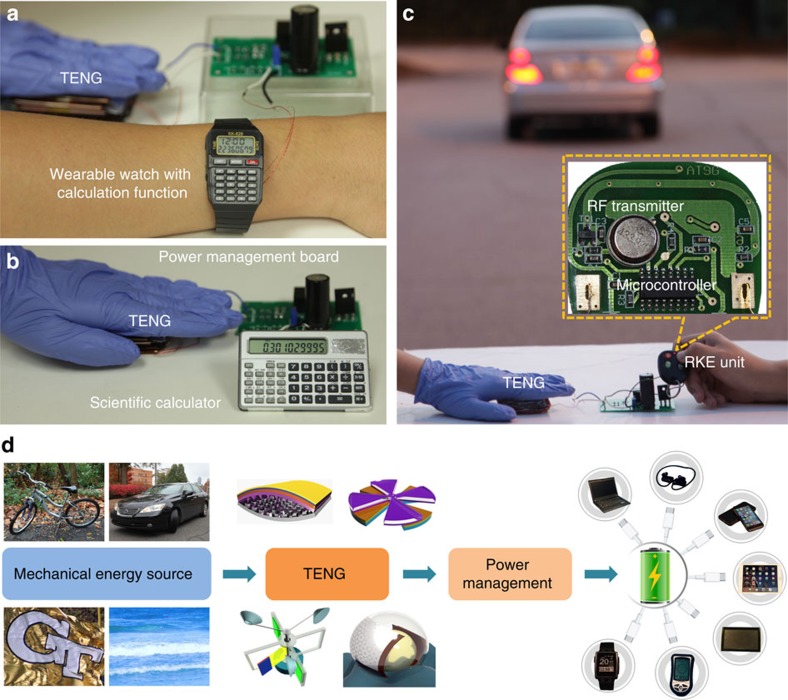
Application in self-powered data-processing and transmission system. (**a**) Demonstration of a self-powered wearable watch and calculator. (**b**) Demonstration of a self-powered scientific calculator. (**c**) Demonstration of a self-powered RKE system. (**d**) Extension of this TENG-based self-charging unit for various applications as a universal adaptable power source.

Besides the self-powered human-activity sensors, this kind of mechanical power unit also has broad applications for other personal electronics. First, its application in wearable electronics is demonstrated through a self-powered wearable watch with mathematical calculation function (Fig. 4a, detailed system diagram shown in Supplementary Fig. 16). As shown in Supplementary Movie 4, a very slow and gentle palm tapping (1 Hz) could drive this wearable watch with full functionality: accurately recording time and calculating square root as an example. Under even irregular palm tapping (have irregular interval between the palming), this wearable watch still remains its normal function (Supplementary Movie 8) as long as the average power provided by human motion is larger than its power consumption. In addition, more complicated data-processing work can also be finished with the power provided by palm tapping. For example, a scientific calculator is a commonly used complicated data-processing system, containing clock generators, registers, read-only memories, arithmetic logic units and a LCD display (Fig. 4b, detailed system diagram shown in Supplementary Fig. 17). As shown in Supplementary Movie 5, such an advanced scientific calculator could be easily powered by the mechanical energy from palm tapping. All of the advanced calculation such as trigonometric function, exponent function and logarithm function could be realized without existence of any batteries. Finally, this power unit is applicable in wireless communication system as well. As a typical demo, a remote keyless entry (RKE) module was connected with this power unit and utilized to wirelessly control a car that was about 50 m away. The RKE module is a complicated radio-frequency system, mainly containing a microcontroller and a radio-frequency transmitter (Fig. 4c, detailed system diagram shown in Supplementary Fig. 18). Once the button on the RKE module is pressed, the microcontroller processes and encrypts the signal and transfers the encrypted 64–128-bit data to the radio-frequency transmitter. Then the 433.92 MHz radio-frequency transmitter can send out the code remotely with a data transfer rate about 2–20 kHz. As shown in Supplementary Movie 6, this power unit could charge the storage capacitor from 5.9 to 6.4 V in about 3–5 s. Once the storage capacitor's voltage reached 6.4 V, this RKE unit could functionalize and send out the encrypted signal and the storage capacitor's voltage went back to about 5.9–6.2 V. After receiving this radio-frequency signal, the receiver inside the car could process, decode and respond to this command (unlocking its door and lighting up its low-beam light), clearly showing the success of remote wireless transmission. Then the storage capacitor was charged by this power unit to reach 6.4 V again. From Supplementary Fig. 19, this self-powered wireless remote system has enough power to send out signals to 50 m away in a speed as fast as a command in every 3.06 s.

## Discussion

The demos presented above have covered all of the fundamental parts of mobile and wearable systems, including sensors, microcontrollers, memories, arithmetic logic units, displays and even wireless transmitters, which have broad applications in personal sensor systems and internet of things. Besides human biomechanical energy, this kind of designs and concepts can also be extended to other mechanical energy sources, such as rolling wheels, moving cars and trains, blowing wind, and surging waves, by applying various available TENG designs. If high-frequency mechanical agitations are utilized to drastically improve the TENG output power, such system will have a potential to serve as a universal standard power source for sustainably driving more complicated electronic systems, including smart watches, cell phones, navigation system, tablets, personal computers and sensor nodes in internet of things (Fig. 4d).

In summary, we have developed a universal self-charging system driven by random body motion for sustainable operation of mobile and wearable electronics. This power unit can provide a continuous d.c. output as driven by low-frequency (∼2 Hz) human biomechanical energy that could be random in amplitude and frequency, which is enough for sustainable operation of many mobile electronics, such as temperature sensors, heart rate monitoring devices, pedometers, wearable watches, scientific calculators and radio-frequency wireless transmitters. This self-charging unit is a paradigm shift towards infinite-lifetime energy sources that can never be achieved solely by batteries. The concept proposed in this paper can also be easily extended into other energy harvesters based on piezoelectric and pyroelectric effects. This study overcomes a bottleneck problem towards self-powered systems, which can have broad applications in mobile/wearable electronics, internet of things, remote environmental monitor devices and wireless sensor networks.

## Methods

### Fabrication of nanostructure on FEP surface

A piece of 125-μm FEP film is rinsed with menthol, isopropyl alcohol and deionized water. Then a 10-nm-thick Au is sputtered onto the FEP surface, which will act as the mask for the etching process. Subsequently, this FEP is etched through the inductively coupled plasma (ICP) reactive ion etching for 60 s. The reaction gas is 15.0 sccm Ar, 10.0 sccm O_2_ and 30.0 sccm CF_4_ in the ICP process. The two ICP power is 400 and 100 W, respectively^39^.

### Fabrication of nanostructure on Al foil

A piece of 50-μm Al foil is pretreated with 0.125 mol l^−1^ NaOH solution at 40 °C for 1 min. Then the Al foil is rinsed with deionized water, and then used as anode and put into the 80-°C etching solution (3 mol l^−1^ H_2_SO_4_ and 1 mol l^−1^ HCl (3:1)). Graphite electrode plates were utilized as cathode. A constant current density of 150 mA cm^−2^ is applied to realize anode oxidation^40^.

### Fabrication of multilayered triboelectric nanogenerators

A 125-μm Kapton film (2.2 inch width) is shaped into a zigzag structure with 10–15 layers. In all, 10–15 layers of aluminium foil and FEP layer are prepared and etched as triboelectric materials. 30 nm Cr/ 300 nm Cu is then e-beam evaporated at the backside of FEP layer. Then these aluminium foils and FEP layers is attached to the substrate. Note that on the front side surface and the backside surface of one layer of Kapton, it must be attached to the same material (Al foil or FEP) to minimize the unnecessary parasitic capacitance that will hurt the performance.

## Additional information

**How to cite this article:** Niu, S. *et al*. A Universal self-charging system driven by random biomechanical energy for sustainable operation of mobile electronics. *Nat. Commun.* 6:8975 doi: 10.1038/ncomms9975 (2015).

## Supplementary Material

Supplementary Information Supplementary Figures 1-20, Supplementary Table 1, Supplementary Discussion and Supplementary References

Supplementary Movie 1 Self-powered temperature sensor continuously driven by palm tapping.

Supplementary Movie 2 Self-powered heart-rate sensor continuously driven by palm tapping.

Supplementary Movie 3 Self-powered pedometer continuously driven by palm tapping.

Supplementary Movie 4 Self-powered wearable electronics continuously driven by palm tapping.

Supplementary Movie 5 Self-powered scientific calculator continuously driven by palm tapping.

Supplementary Movie 6 Self-powered wireless communication system continuously driven by palm tapping.

Supplementary Movie 7 Self-powered pedometer continuously driven by foot tapping.

Supplementary Movie 8 Self-powered wearable electronics continuously driven by irregular palm tapping.
