# Supplementary material for: A universal self-charging system driven by random biomechanical energy for sustainable operation of mobile electronics
Source: Nat Commun. 2015 Dec 11;6:8975. doi: 10.1038/ncomms9975 (PMC4682168; doi:10.1038/ncomms9975)
Supplement: Supplementary Information — Supplementary Figures 1-20, Supplementary Table 1, Supplementary Discussion and Supplementary References [file ncomms9975-s1.pdf]

Supplementary Figures:

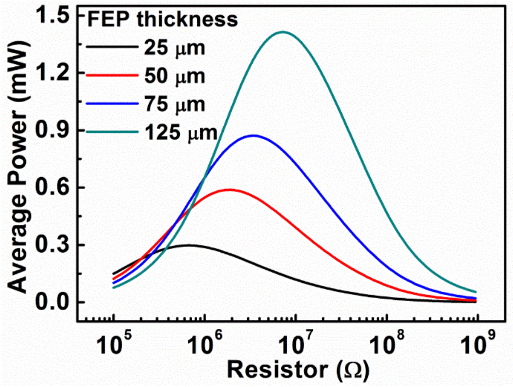

Supplementary Figure 1. Theoretical calculation results to optimize the FEP layer thickness

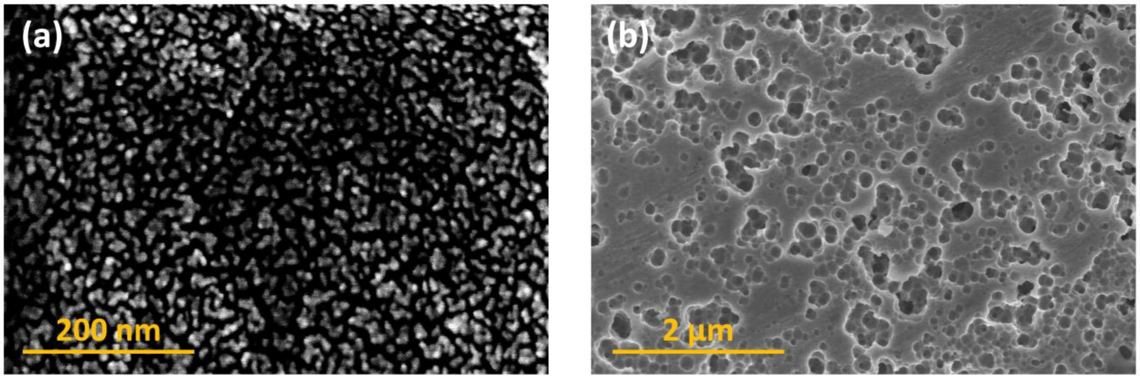

Supplementary Figure 2. SEM picture of the surface of (a) FEP (b) Al foil

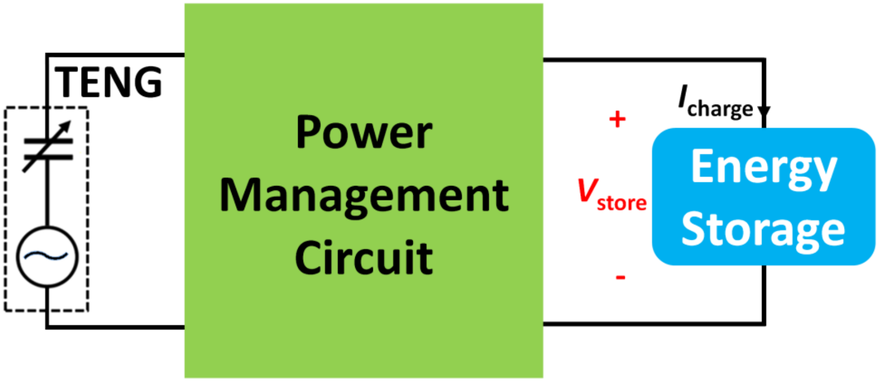

Supplementary Figure 3. Simulation circuit diagram for theoretical calculation of $P_{store,avg}$

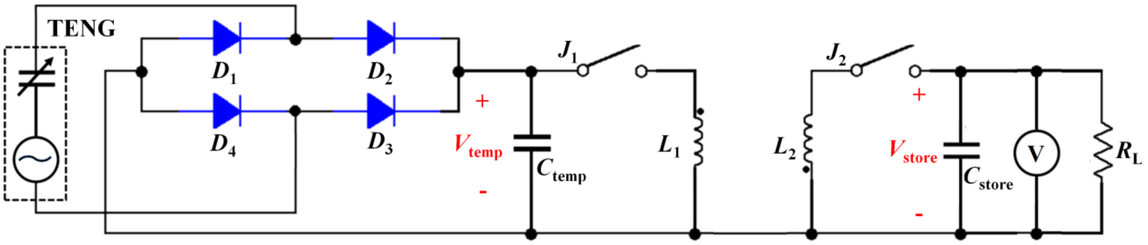

Supplementary Figure 4. Experiment measurement matrix for $P_{store,avg}$

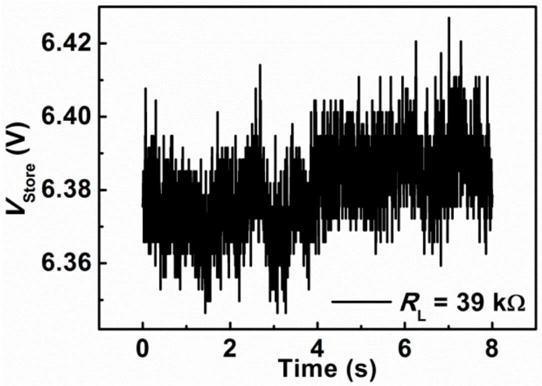

Supplementary Figure 5. Enlarged figure of the curve shown in Figure 2d when R_L equals to 39 kΩ

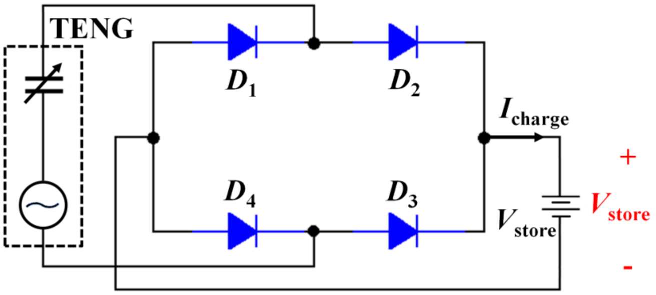

Supplementary Figure 6. Circuit diagram for direct charging

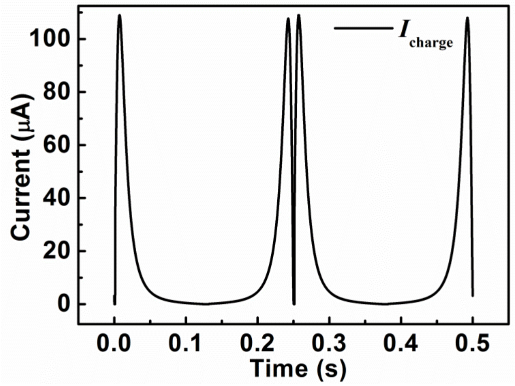

Supplementary Figure 7. Simulated charging current profile for direct charging of a 1 V ideal battery

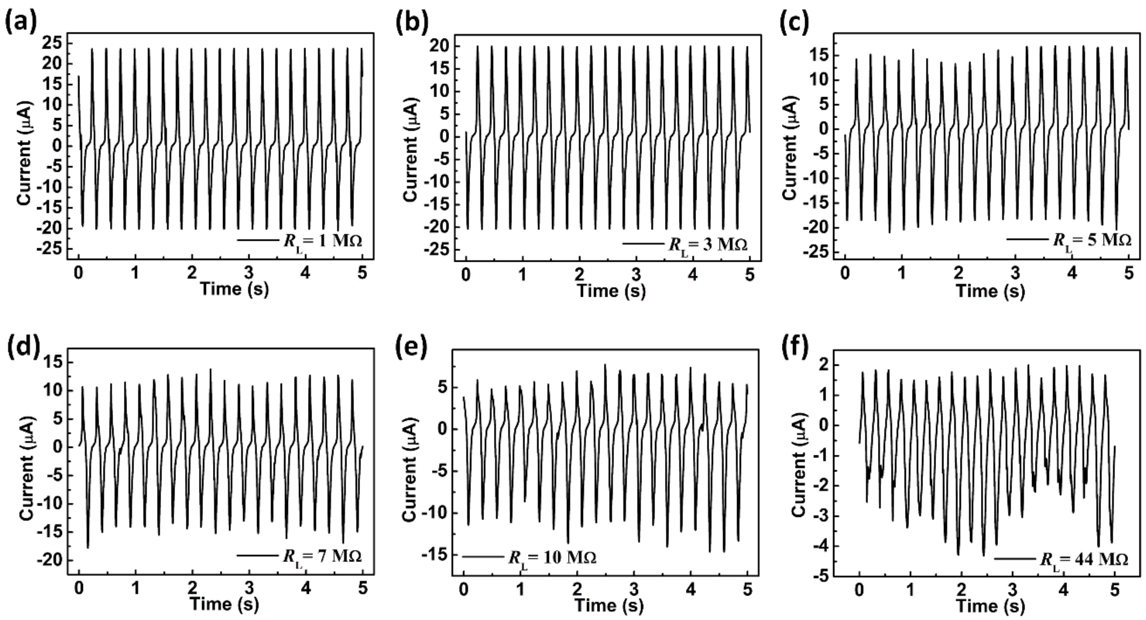

Supplementary Figure 8. Measured AC Current profile of different load resistors. (a) 1 MΩ load resistor. (b) 3 MΩ load resistor. (c) 5 MΩ load resistor. (d) 7 MΩ load resistor. (e) 10 MΩ load resistor. (f) 44 MΩ load resistor.

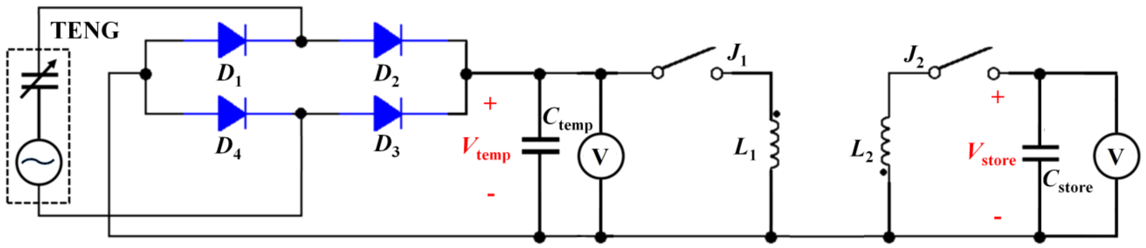

Supplementary Figure 9. Experimental measurement matrix for board efficiency

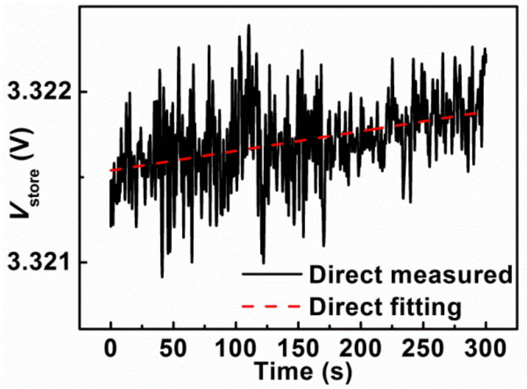

Supplementary Figure 10. Enlarged figure of the direct charging curves shown in Figure 2g

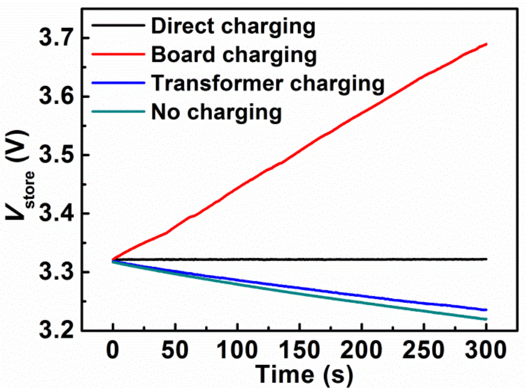

Supplementary Figure 11. Comparison of direct charging, board charging and transformer charging.

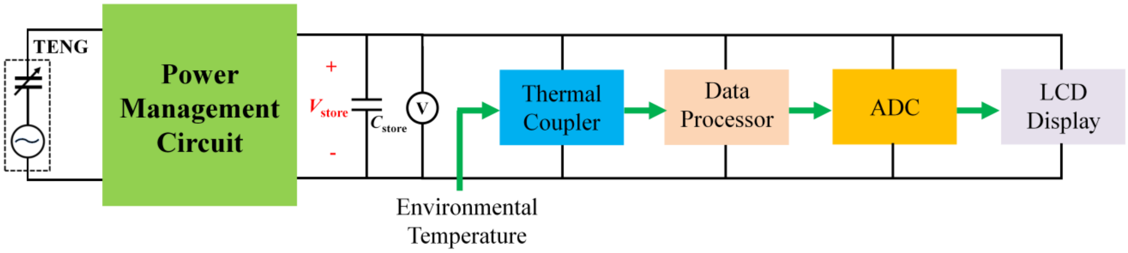

Supplementary Figure 12. System diagram of self-powered temperature sensor application

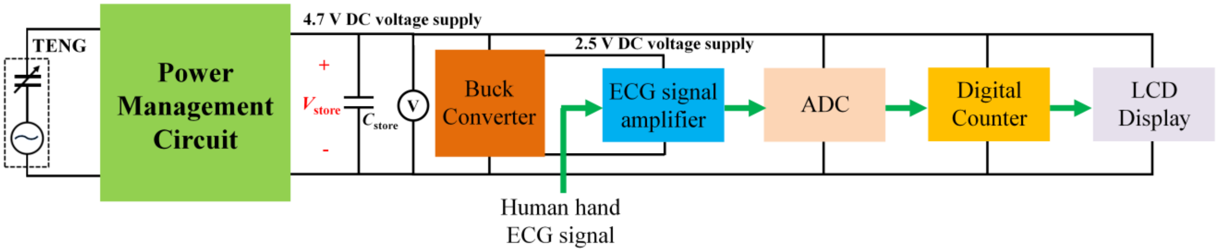

Supplementary Figure 13. System diagram of the self-powered ECG system

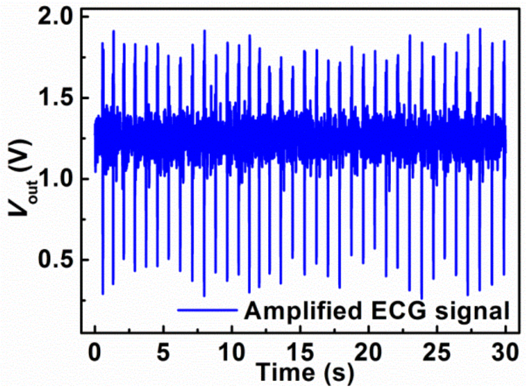

Supplementary Figure 14. Amplified heart rate signal through the ECG signal amplifier

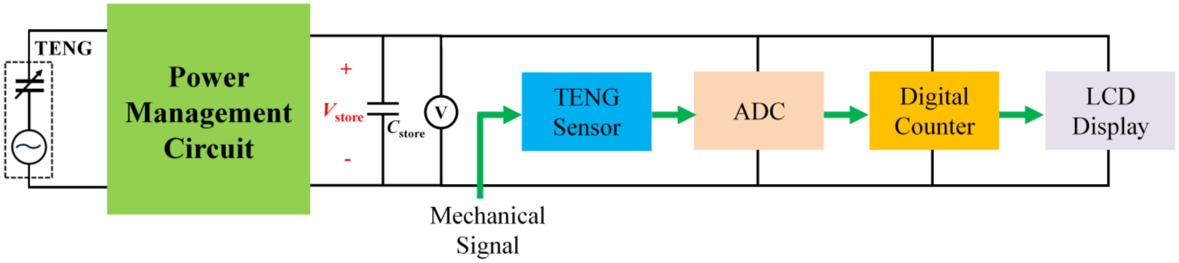

Supplementary Figure 15. System diagram of the self-powered pedometer system

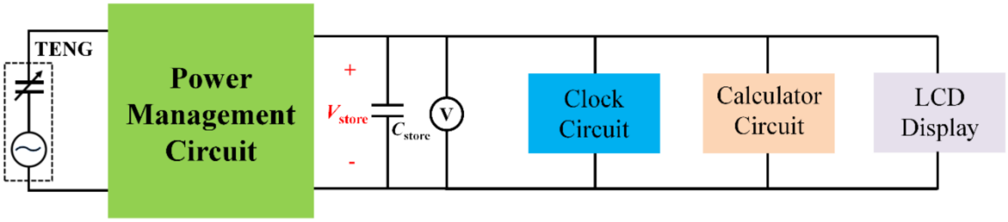

Supplementary Figure 16. System diagram of the self-powered wearable watch and calculator system

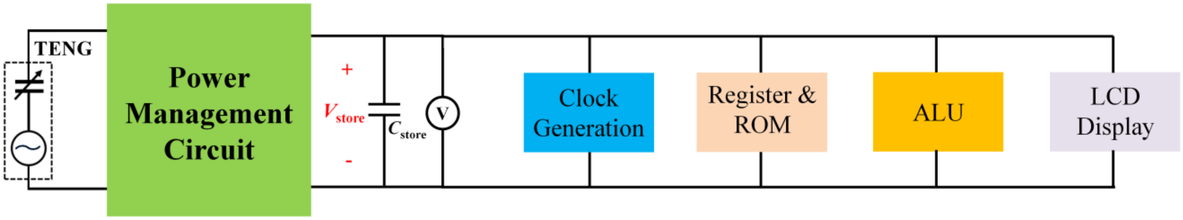

Supplementary Figure 17. System diagram of the self-powered scientific calculator system

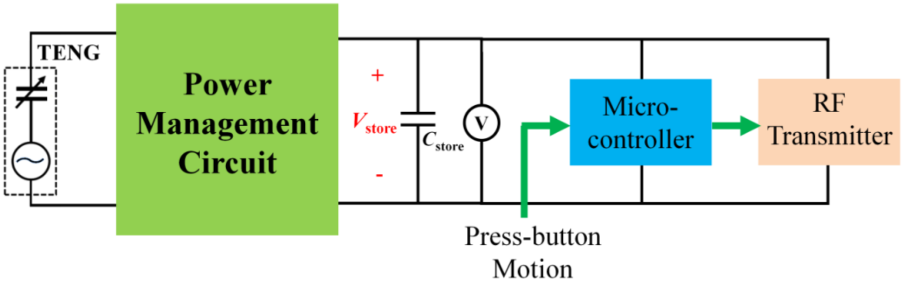

Supplementary Figure 18. System diagram of the self-powered RKE unit

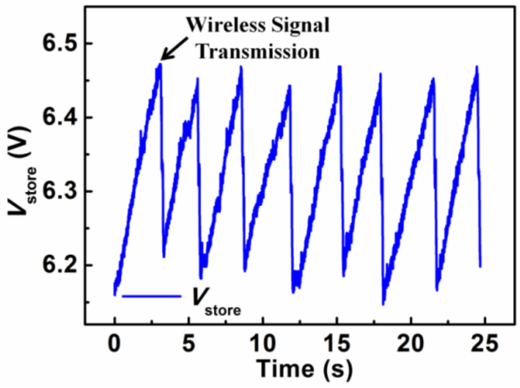

Supplementary Figure 19. Voltage profile of the storage capacitor when the RKE unit is in function

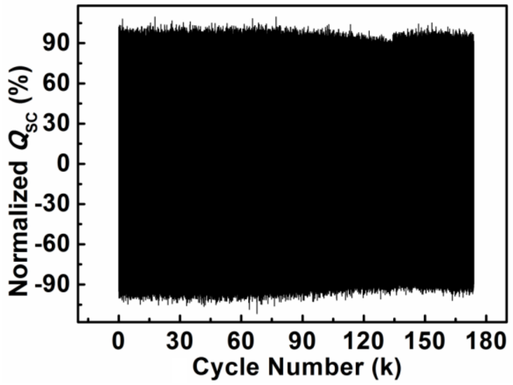

Supplementary Figure 20. Durability test of the as-fabricated multilayer TENG.

Supplementary Table:

FEP layer thickness d_r	25/50/75/125 μm
FEP layer dielectric constant ε_r	2
Area size of Dielectrics S	2.2 inch * 2 inch *15
Tribo-charge surface density σ	50 μCm^{-2}
Air gap thickness g	(1.35- d_r/1000) mm
Period of external motion T	0.25 s

Supplementary Table 1. Parameters utilized for theoretical simulation of TENG output

Supplementary Discussion:

1. Theoretical optimization of FEP layer thickness

We optimize the FEP layer thickness by maximizing the average power output under resistive loads. The total thickness for one layer of the multilayered TENG is measured to be about 1.6 mm (1.6 cm for a 10-layer TENG and 2.4 cm for a 15-layer TENG) due to the elastic property of the Kapton substrate. For each layer, the combined thickness of the Al foil and the supporting Kapton substrate is about 250 μm. So if the FEP layer thickness is d_r μm, the thickness of the air gap will be (1.35- d_r/1000) mm. Besides, since thick FEP film results in curved FEP surface, the maximum FEP layer thickness we choose is the same with the Kapton substrate (125 μm).

The detailed theoretical calculation parameter is shown in Supplementary Table 1. In the theoretical calculation, we utilize the simplest motion: a harmonic vibration as shown in Supplementary Equation 1. (The *x* in the following equation represents the separation distance of the top surface FEP and the Al foil.)

$$x(t) = \frac{x_{\max}}{2}\left[1 - \cos\left(\frac{2\pi t}{T}\right)\right] \qquad (1)$$

For an attached-electrode contact-mode TENG connected with a resistive load *R*, the government equation for the whole system can be given by: [1]

$$R\frac{\mathrm{d}Q}{\mathrm{d}t} = -\frac{d_0 + x(t)}{\varepsilon_0 S}Q + \frac{\sigma x(t)}{\varepsilon_0} \qquad (2)$$

In Supplementary Equation 2, the effective dielectric thickness d_0 can be defined as: [1]

$$d_0 = \frac{d_\mathrm{r}}{\varepsilon_\mathrm{r}} \qquad (3)$$

To solve the steady state of this first-order differential equation, we can apply the periodic boundary condition as:

$$Q(t = 0) = Q(t = T) \qquad (4)$$

And once the steady-state solution for *Q* is obtained, we can calculate the steady-state current output by:

$$I(t) = \frac{\mathrm{d}Q}{\mathrm{d}t} \qquad (5)$$

The average power $P_{\mathrm{R,avg}}$ delivered to the load *R* can be calculated as:

$$P_{\mathrm{R,avg}} = \frac{1}{T}\int_0^T I(t)^2 R\mathrm{d}t \qquad (6)$$

The average power output under different d_r and R is calculated, as shown in Supplementary Figure 1. From Supplementary Figure 1, among the 4 available FEP films, the 125 μm FEP layer can provide the maximized average power. In this parameter set, it is close to 1.41 mW.

2. SEM pictures of nanostructure on the surface of FEP layer and Al foil

The SEM pictures of nanostructure on the surface of FEP layer and Al foil are shown in Supplementary Figure 2a and b, respectively.

3. Definition of total efficiency η_{total} and measurement matrix for the total efficiency

The total efficiency η_{total} is defined as the ratio of the power that can be stored into the storage element ($P_{store,avg}$) to the maximum average AC power that can be harvested on a resistive load ($P_{R,avg,opt}$), which can be given by:

$$\eta_{\text{total}} = \frac{P_{\text{store,avg}}}{P_{\text{R,avg,opt}}} \qquad (7)$$

3.1 Theoretical calculation and experimental measurement of $P_{R,avg,opt}$

Theoretically, the maximized average AC power that can be harvested on a resistive load can be calculated in the same procedure shown above (Supplementary Equation 2 to 6) to obtain the $P_{R,avg} – R$ curve. Once $P_{R,avg} – R$ curve has been obtained, $P_{R,avg,opt}$ can be obtained as the maximized value for $P_{R,avg}$ on the matched load resistance.

Experimentally, $P_{R,avg,opt}$ can be measured through the following method. First, the TENG is under mechanical motion provided by an electric motor to ensure the stability of the external motion. Then, the TENG is in serial connected with a load resistance and a current meter (SR570, Stanford Research Systems) to measure the current through load resistance. Through the current signal, we can calculate $P_{R,avg}$ under this load resistance. By changing the load resistance to different values, an experimental $P_{R,avg}$ – R curve can be obtained through interpolation method. Finally, $P_{R,avg,opt}$ can be obtained as the maximized value for $P_{R,avg}$ from the interpolated $P_{R,avg} – R$ curve.

3.2 Theoretical calculation and experimental measurement of $P_{store,avg}$

Theoretically, $P_{store,avg}$ can be simulated through TENG simulator.[2] The simulated circuit diagram can be shown in Supplementary Figure 3.

From TENG simulator, the V_{store} and I_{charge} information can be easily obtained and then $P_{\text{store,avg}}$ can be calculated as:

$$P_{\text{store,avg}} = \frac{1}{T}\int_0^T V_{\text{store}}(t) I_{\text{charge}}(t)\mathrm{d}t \qquad (8)$$

Experimentally, since direct measurement of the I_{charge} peak signal results in large measurement error, another measurement method is utilized. In the experiment, we utilize a 1mF Al-electrolyte capacitor (C_{store}) as the energy storage unit and add a load resistor R_L in parallel with C_{store}, as shown in Supplementary Figure 4.

Under this circuit configuration, the storage capacitor stores the energy coming from TENG and sends out the energy to the load resistance R_L. When R_L is large, the energy consumed by R_L is little, so there is still extra energy from TENG to be stored into the C_{store}. Therefore, the voltage of the storage capacitor V_{store} rises up with time and a positive slope of $V_{\text{store}} - t$ curve can be observed from a voltage meter that is in parallel with C_{store} (Keithley 6514). When R_L decreases, the energy consumed by R_L increases and is gradually approaching the energy that TENG can provide. When the energy provided from TENG equals to the energy consumed by R_L, the $V_{\text{store}} - t$ curve will have a 0 slope. So to calculate $P_{\text{store, avg}}$, an easy method is that we change the load resistance R_L and measure the corresponding $V_{\text{store}} - t$ curve. Once the slope of $V_{\text{store}} - t$ curve is close to 0, the power consumed by R_L is equal to $P_{\text{store, avg}}$. At this time, we can obtain the average value of V_{store} from the measured $V_{\text{store}} - t$ curve and $P_{\text{store, avg}}$ can be given by:

$$P_{\text{store,avg}} = \frac{{V_{\text{store,avg}}}^2}{R_L} \qquad (9)$$

In the measurement of the DC power delivered to the load (Figure 2d, enlarged figure shown in Supplementary Figure 5), we connect two 15-layer multilayered TENG in parallel as the energy source and use a 1 mF Al-electrolyte capacitor as the energy storage unit. The measured DC power under palm tapping is 1.044 mW (7.34 Wm^{-3}).

3.3 Theoretical calculation results of η_{total} for a direct charging of a 1 V battery

For direct charging, the power management circuit just contains 4 diodes. Supplementary Figure 6 shows the whole circuit diagram for this case. Inside the circuit diagram, the battery is idealized as an ideal voltage source and its internal resistance and leakage current are neglected. Besides, the leakage current of the diode utilized in the simulation are all ultralow to ensure the maximized charging efficiency.

The charging performance of direct charging can be easily simulated from TENG simulator.[2] The TENG simulation parameter is the same as listed in Supplementary Table 1 and the external motion process is the same as shown in Supplementary Equation 1. The simulated charging current profile is shown as in Supplementary Figure 7.

Through Supplementary Equation 8, the average charging power can be calculated as 16.5 μW. Compared to the simulated resistive load AC power (1.41 mW), the theoretical charging efficiency is only 1.17% even the unideal factors of the battery (the leakage current and the internal resistance) are all neglected.

3.4 Theoretical calculation results of η_{total} for charging a capacitor

When an attached-electrode TENG is utilized to charge a capacitor C_{temp} from 0 V, the charging curve can be shown as:[3]

$$V_{\text{temp}} = V_{\text{sat}}\left[1 - \exp\left(-\frac{kft}{C_{\text{temp}}}\right)\right] \qquad (10)$$

Inside the equation,[1,3]

$$V_{\text{sat}} = \frac{\sigma g d_0}{\varepsilon_0(g + 2d_0)} \qquad (11)$$

$$k = \frac{2\varepsilon_0 S(g + 2d_0)}{d_0(d_0 + g)} \qquad (12)$$

We have derived the optimum charging time t_{opt} equals to $1.25643C_{\text{temp}}/(kf)$ and the optimum charging voltage $V_{\text{c,opt}}$ equals to $0.7153V_{\text{sat}}$. So the theoretically maximized charging power can be calculated as:

$$P_{\text{store,avg,opt}} = \frac{C_{\text{temp}}{V_{\text{opt}}}^2}{2t_{\text{opt}}} = 0.40723fS\frac{\sigma^2 g^2 d_0}{\varepsilon_0(g + 2d_0)(g + d_0)} \qquad (13)$$

Utilizing the parameters listed in Supplementary Table 1, $P_{\text{store,avg,opt}}$ can be calculated as 1.057 mW. Compared to the average AC power that the TENG can provide (1.41 mW), this working principle can provided a maximum η_{total} equal to 75.0%, which is huge improvement compared with the direct charging efficiency.

If the capacitor charging cycle doesn't start from 0 V, from theoretical calculation, the maximum $P_{\text{store,avg}}$ can be reached when the capacitor starts charging at 0.5 V_{sat} and sends out the energy at 0.5 V_{sat}. At this time, the maximized $P_{\text{store,avg}}$ is $0.5fS\frac{\sigma^2 g^2 d_0}{\varepsilon_0(g+2d_0)(g+d_0)}$, only a 23% improvement from charging from 0 V.

However, reaching this charging condition requires ultrahigh speed of logic control circuit and switch, which will also increase the power consumption and reduce the board efficiency. So in this design, we still choose to charge the capacitor from a value close to 0.

3.5 Calculation of η_{total} from experimental data

Utilizing the above experimental procedure, we first calculate $P_{R,avg,opt}$. A 10-layer TENG is utilized in this measurement and a 4 Hz stable mechanical motion is generated through an electricity-driven shaker. The measured current profile for different load resistors is shown as Supplementary Figure 8.

Through Supplementary Equation 6, the average power for these load resistance can be calculated. Then continuous fraction interpolation is utilized to obtain the $P_{R,avg} - R$ curve. From the interpolated curve (shown in Figure 2e), $P_{R,avg,opt}$ is calculated to be 0.3384 mW under a 4.26 MΩ matched load resistance.

Second, we can experimentally measure $P_{store,avg}$. The experimental procedure is the same as we mention above. When the load resistance is 180 kΩ, the slope of V_{store}- t curve is still positive but close to 0. From the measured V_{store}- t curve shown in Figure 2f, $V_{store,avg}$ is 6.0343 V and $P_{store,avg}$ is calculated to be 0.2023 mW from Supplementary Equation 9. So experimentally, η_{total} is calculated to be 59.8%.

4. Energy transfer efficiency by directly transfer energy from a small capacitor and a large capacitor

Energy transfer directly from a small capacitor to a large capacitor will result in ultralow efficiency. We consider a general case that we transfer energy from a small capacitor C with an initial voltage V to a large capacitor kC with an initial voltage 0.

At the initial stage, the total energy inside the system is shown as:

$$E_{\text{total,initial}} = \frac{1}{2}CV^2 + \frac{1}{2}kC \times 0^2 = \frac{1}{2}CV^2 \quad (14)$$

After the energy transfer, the small capacitor and the large capacitor will have the same voltage V'. Due to the charge conservation, the relationship between V' and V can be given by:

$$(kC + C)V' = CV \quad (15)$$

Therefore,

$$V' = \frac{V}{k+1} \quad (16)$$

Therefore, at the final stage, the total energy inside the system can be given by:

$$E_{\text{total,final}} = \frac{1}{2}(kC + C)V'^2 = \frac{1}{2(k+1)}CV^2 \qquad (17)$$

Therefore, the energy transfer efficiency of direct transfer is only 1/(k+1). For the case in our design, k is 1/4.7e-6, which is result in energy transfer efficiency 4.7e-6. The energy loss is from the heat loss through the parasitic resistance inside the system. Therefore, the second stage in the power management circuit is completely necessary to ensure acceptable energy transfer efficiency.

5. Definition of board efficiency and measurement matrix for board efficiency

The board efficiency is for evaluating the energy transfer efficiency from the small capacitor C_{temp} to the large capacitor C_{store}. The definition of the board efficiency is the ratio of the energy sent out from C_{temp} to the energy stored into C_{store}. The experimental measurement matrix for the board efficiency is shown in Supplementary Figure 9. The C_{temp} utilized in this experiment is a 4.7 nF ceramic capacitor and the C_{store} utilized in this experiment is a 1 mF Al-electrolyte capacitor.

Experimentally we can utilize two voltage meters (Keithley 6514) to measure V_{temp} and V_{store} simultaneously. First, we can extract the initial and final value of V_{store} ($V_{\text{store,initial}}$ and $V_{\text{store,final}}$). And the energy stored in C_{store} can be calculated as:

$$E_{\text{store}} = \frac{1}{2}C_{\text{store}}({V_{\text{store,final}}}^2 - {V_{\text{store,initial}}}^2)$$

From the measurement data shown in Figure 2b, the initial and final value of V_{store} is and 4.99038 V and 6.43343 V. So the energy stored in C_{store} can be calculated to be 8.243 mJ.

To extract the energy sent out from C_{temp}, we can extract all the peaks when V_{temp} suddenly falls off. Through data processing, the total 249 peaks can be extracted with the initial and final value of V_{temp} every time when V_{temp} falls off. Then the energy sent out from C_{temp} can be calculated as

$$E_{\text{sent}} = \frac{1}{2}C_{\text{temp}}\sum_{i=1}^{249}({V_{\text{temp,initial,i}}}^2 - {V_{\text{temp,final,i}}}^2)$$

From the measurement data shown in Figure 2c, the energy sent out from C_{temp} can be calculated to be 9.160 mJ. So the board efficiency can be calculated to be 90.0%.

6. Comparison of charging characteristics among direct charging, transformer charging, and board charging

For comparison of different charging method, we utilize the same TENG (a 10-layer multi-layer TENG) under the same mechanical motion (6Hz, from an electric-driven shaker) to charge the same supercapacitor (12mF, Cellergy CLG06P012L12). The circuit diagram for board charging is the same as shown in Supplementary Figure 9 and the circuit diagram for direct charging is the same as shown in Supplementary Figure 6.

We measure the voltage on the supercapacitor through the voltage meter (Keithley 6514). Then we can linear fitting the measured voltage-time curve to extract the slope k. From the data measured, the slope of board charging curve is 1.2613 mV/s, so the average charging current can be calculated as 15.14 μA. The slope of direct charging curve is 1.1514 μV/s (enlarged curve shown in Supplementary Figure 10) and the corresponding average charging current is 13.82 nA. The average charging current from the power management board is 1095.4 times larger than direct charging for this case.

Besides the comparison of board charging and direct charging, we also charge the same supercapacitor with a 10:1 transformer and measure the direct leakage current of this supercapacitor, as shown in Supplementary Figure 11. In this case, if a transformer is utilized, the average charging current cannot even compensate the leakage current, which again proves the low efficiency of transformer charging in harvesting low-frequency biomechanical energy.

7. Demo 1: Self-powered temperature monitoring system

The whole self-powered system is shown in Supplementary Figure 12. In the whole system, there are no functional components that need external electrical power source. A 15-layer TENG is utilized as the energy harvesting device and a 1mF Al-electrolyte capacitor is utilized. To eliminate the confusion of load energy supply, a battery is not suitable here. (If a battery is utilized, since the voltage of a battery keeps constant during the charging and discharging process, it is not known whether the energy consumed by the load circuit is from the battery residue energy or from TENG. For a capacitor, the energy stored is proportional to the voltage. Therefore, if the voltage of the storage capacitor doesn't drop, the load energy supply must come from the harvested mechanical energy.) Besides the functional part, a voltage meter (Keithley 6514) is utilized to monitor the voltage of the storage capacitor and to clarify the possible confusion that the power supply may be provided from the stored energy of the storage capacitor. If the voltage of the storage capacitor doesn't drop from its initial value, the energy consumed by the functional circuit is completely provided by the TENG through the power management circuit.

The detailed demo is shown in Supplementary Movie 1. We utilize this temperature sensor to measure the room temperature, human finger temperature, cold water temperature, and hot water temperature. The voltage of the storage capacitor is always greater than its initial value, showing that the power generated from this system is enough for the temperature sensor.

8. Demo 2: Self-powered ECG system

We still utilize a 1mF Al-electrolyte capacitor as the storage element and a voltage meter to monitor the storage capacitor. However, because of the relatively large power consumption of this system, two as-fabricated 15-layer TENGs are connected in parallel as the power source. The whole system diagram is shown in Supplementary Figure 13.

Inside the system, the LCD display requires its supplying voltage to be larger than 4.5 V, but the ECG signal amplifier requires its supplying voltage to be smaller than 3.5 V. Therefore, a buck converter is added to efficiently convert a 4.7 V DC voltage supply from the storage capacitor to a 2.5 V DC voltage supply. The bioelectricity from human hand is collected by two Al metal rods. And this collected bioelectricity is amplified and filtered through the ECG signal amplifier. The amplified human heart rate signal is shown in Supplementary Figure 14. The amplified heart rate signal can be digitalized through an analog-to-digital converter (ADC) and then digitalized heart rate signal is captured by a digital counter. So once there is a heart rate peak, the output of the digital counter will increase by 1. Finally, the output of the digital counter is visualized through a LCD display with its decoder.

9. Demo 3: Self-powered pedometer system

In this self-powered pedometer system, we still utilize one 15-layer TENG as the energy harvesting device, a 1mF Al-electrolyte capacitor as the storage element, and a voltage meter to monitor the storage capacitor. The whole system diagram is shown in Supplementary Figure 15.

Inside this system, a TENG-based self-powered mechanical sensor is utilized to minimize the power consumption. This TENG is also an attached-electrode contact-mode TENG, but only contains one layer (as shown in Figure 1b). The triboelectric pair material in the TENG is Al and FEP. Al is utilized as one electrode and at the backside of the FEP, copper is deposited as another electrode. The whole structure is supported by two acrylic sheets and an elastic spring is utilized to separate these two acrylic sheets. When this TENG sensor is under pressure and then released, it will generate a voltage peak signal that is close to the amplified ECG signal above. That peak can be easily captured by the ADC and the digital counter.

10. Demo 4: Self-powered wearable watch & calculator system

The whole system diagram is shown in Supplementary Figure 16. The TENG and energy storage device is the same as above demos.

11. Demo 5: Self-powered scientific calculator system

The whole system diagram is shown in Supplementary Figure 17. The TENG and energy storage device is the same as above demos.

In Supplementary Movie 5, we demonstrate the normal function of this circuit by calculating the following 3 equations.

$$\sqrt{5} \approx 2.236067977 \quad \sin 60^{\circ} \approx 0.866025404 \quad \log_{10} 2 \approx 0.301029995$$

12. Demo 6: Self-powered RKE unit

In this self-powered RKE unit, the TENG and energy storage device is the same as above demos. The whole system diagram is shown in Supplementary Figure 18.

This RKE unit mainly contains a microcontroller together with a RF Transmitter. After the button is pressed, the microcontroller will generate a 64-128 bit code that containing a data preamble, a command code, some check bits, and a "rolling code" that ensures vehicle security by altering itself with each use. Then this 64-128 bit code is sent out through the RF transmitter. The carrier frequency of the RF transmitter is 433.92 MHz and its data sending rate is 2 - 20 KHz. This fast data sending rate can ensure the sending time to be less than 64 ms. When this RKE unit is connected with the self-charging power unit, the self-charging power unit can charge the 1 mF storage capacitor from 5.9 V to 6.4 V in about 3-5 seconds. Once the storage capacitor’s voltage reaches 6.4 V, the RKE unit can functionalize and send out the encrypted signal and the storage capacitor’s voltage goes back to about 5.9-6.2 V. Such working pattern of this self-powered RKE unit is shown in Supplementary Figure 19. From Supplementary Figure 19, the self-powered RKE unit can send out an encrypted command in every 3.06 seconds without any external electrical power supply. After receiving the code, a car that is 50 meters away can unlock its front door and light up its front and rear position light, which clearly shows the normal function of this RKE unit.

13. Durability test of the TENG's performance

To test the durability of the device, we use an electricity-driven motor to hit the device for about 180,000 cycles and monitor Q_{SC} output of the device. As shown in Supplementary Figure 20, the durability of the device is very good and Q_{SC} does not show obvious degradation even after 180,000 cycles.

Supplementary References:

1 Niu, S. M. *et al.* Theoretical study of contact-mode triboelectric nanogenerators as an effective power source. *Energy Environ. Sci.* **6**, 3576-3583, (2013).

2 Niu, S. M. *et al.* Simulation method for Optimizing the performance of an integrated triboelectric nanogenerator energy harvesting system. *Nano Energy* **8**, 150-156, (2014).

3 Niu, S. M. *et al.* Optimization of charging performance in triboelectric nanogenerators for efficient energy harvesting *IEEE Trans. Electron Devices* **62**, 641-647, (2015).
